# Anomalous Thermal Response of Graphene Kirigami Induced by Tailored Shape to Uniaxial Tensile Strain: A Molecular Dynamics Study

**DOI:** 10.3390/nano10010126

**Published:** 2020-01-09

**Authors:** Hui Li, Gao Cheng, Yongjian Liu, Dan Zhong

**Affiliations:** 1 School of Highway, Chang’an University, Xi’an 710064, China; leehui@chd.edu.cn; 2 Zhuhai Da Hengqin Science and Technology Development Co. Ltd., Zhuhai 519000, China

**Keywords:** graphene kirigami, mechanical properties, thermal conductivity, molecular dynamics

## Abstract

The mechanical and thermal properties of graphene kirigami are strongly dependent on the tailoring structures. Here, thermal conductivity of three typical graphene kirigami structures, including square kirigami graphene, reentrant hexagonal honeycomb structure, and quadrilateral star structure under uniaxial strain are explored using molecular dynamics simulations. We find that the structural deformation of graphene kirigami is sensitive to its tailoring geometry. It influences thermal conductivity of graphene by changing heat flux scattering, heat path, and cross-section area. It is found that the factor of cross-section area can lead to four times difference of thermal conductivity in the large deformation system. Our results are elucidated based on analysis of micro-heat flux, geometry deformation, and atomic lattice deformation. These insights enable us to design of more efficient thermal management devices with elaborated graphene kirigami materials.

## 1. Introduction

Strain engineering can effectively regulate the thermal conductivity of traditional bulk materials [1,2,3] as well as low dimensional nanomaterials [4,5,6,7,8]. It has been found that the thermal conductivity of bulk nanostructures increases with increasing compress strain but decreases with increasing tensile strain [1,2,3]. Li et al. reported that the thermal conductivity of bulk Si decreases by 68% when strain increases from −0.09 (compression) to 0.12 (tension) [3]. For low dimensional materials, the thermal properties of graphene [4,5], silicene [6], phosphorene [7], and nanotube [8] are also sensitive to strain engineering. For instance, the thermal conductivity of graphene reduces under uniaxial tensile strain and its maximum reduction reaches 60% at strain of 0.2 [5]. Xu et al. reported that the thermal conductivity of single-walled carbon nanotube is reduced under both tension and compression [8]. Its thermal conductivity reduces by 32% and 30% at strain of −0.06 and 0.15, respectively.

For the most traditional materials, longitudinal deformation usually exhibits little effect on their variation of cross-section area in lateral direction. Thus the effect of the minor variation of cross-section area deriving from longitudinal strain on thermal conductivity is small and can be negligible. It is because the global deformation of these structures actually derives from the microscopic deformation of the atomic bonds and angles, thus the effect of strain on thermal conductivity is mainly attributed to the phonon vibrational density of states, i.e., phonon softening/stiffening and phonon scattering. Compared to traditional materials, there also exist some materials exhibit large deformation capacities under longitudinal loading, the lateral deformation is mainly caused by the geometry deformation. The kirigami graphene structures (KGS) were found to exhibit strong yield and fracture strains than pristine graphene [9,10,11]. Wei et al. studied the effects of tailoring size on mechanical and thermal properties of KGS by introducing rectangular tailoring geometry [11]. Their results show that the thermal response of KGS to uniaxial tensile strain is different from that of pristine graphene. The deformation of KGS under tensile strain is resulting from the competition of the two mechanisms, the geometry deformation and atomic lattice deformation. While, two of them lead to the different thermal response.

When material is conducted uniaxial tensile/compressive strain, it usually deforms in the lateral direction. The Poisson’s ratio is usually used to characterize the relationship between the deformation in the lateral direction for a material and its longitudinal strain. Poisson’s ratio (*v*) is defined as *v* = −*ε_t_*/*ε_l_*, where *ε_l_* and *ε_t_* are the longitudinal strain and the corresponding transverse strain, respectively. Most traditional materials tend to contract in the lateral direction under longitudinal elongation deformation. In contrast, there are also some auxetic materials that tend to expand in the lateral direction, which are called as negative Poisson’s ratio materials, such as single-layer black phosphorene [12], graphene oxide [13], and graphene nanoribbons with small width (<10 nm) [14]. Due to the fact that the magnitude of cross-section area (*A*) is closely related to thermal conductivity of a material, *κ* = *J*/(*A ∂T*/*∂L*), thus Poisson’s ratio could highly affect the thermal conductivity of a material in means of variation value of the cross-section area.

As a typical two-dimensional material, the mechanical property of graphene can be tuned by kirigami. KGS has been realized in experiment using optical lithography [10] and showed a tunable mechanical property. By adjusting tailoring geometry of graphene, kirigami not only improves the deformation capacities of KGS, but also introduces negative Poisson’s ratio properties to KGS [15,16]. To investigate the effect of large deformation on thermal conductivity of materials and further study the difference in structural variation caused by tailoring geometry, we employ three typical KGSs with different tailoring geometry to study their mechanical and thermal properties.

In this paper, we investigate the thermal conductivity of KGSs using molecular dynamics simulations. Three typical KGSs are considered in present study. The effect of tailoring geometry on mechanical property is initially considered. We further choose the two typical kirigami structures, i.e., square kirigami graphene with large deformation capacity, and quadrilateral star structure with negative Poison’s ratio characteristics to explore their structure deformation effects on thermal conductivity.

## 2. Models and Methods

**Figure 1:**
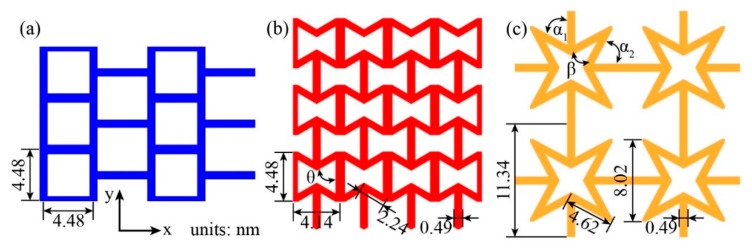
Three models of kirigami graphene structure (KGSs) are used in the present work. (**a**) Square kirigami graphene (SKG) model; (**b**) reentrant hexagonal honeycomb (RHH) model; and (**c**) quadrilateral star structure (QSS).

Three kirigami structures, square kirigami model (SKG), reentrant hexagonal honeycomb (RHH), and quadrilateral star structure (QSS), were considered in the present study. As shown in Figure 1, the geometer parameters are also labeled.

Molecular dynamics simulations were performed to explore the thermal transport properties of KGS using large-scale atomic/molecular massively parallel simulator (LAMMPS) [17]. Intermolecular reactive empirical bond order (AIREBO) potential [18,19] was used to describe carbon–carbon atomic interactions for it could well describe the variation of thermal conductivity of graphene [5,20] and defective graphene [11]. Periodic boundary conditions were applied in both x and y directions. The standard Newton’s equation of motion performed time integration using the Verlet algorithm with a time step of 0.5 femtosecond. The Polak–Ribiere version of the conjugated gradient algorithm [21] was initially adopted to minimize the total energy of system and optimize the structure of system. After that, a 200 ps Nosé–Hoover thermal bath coupling [22,23] is conducted to ensure the system reaches the equilibrium state at 300 K.

**Figure 2:**
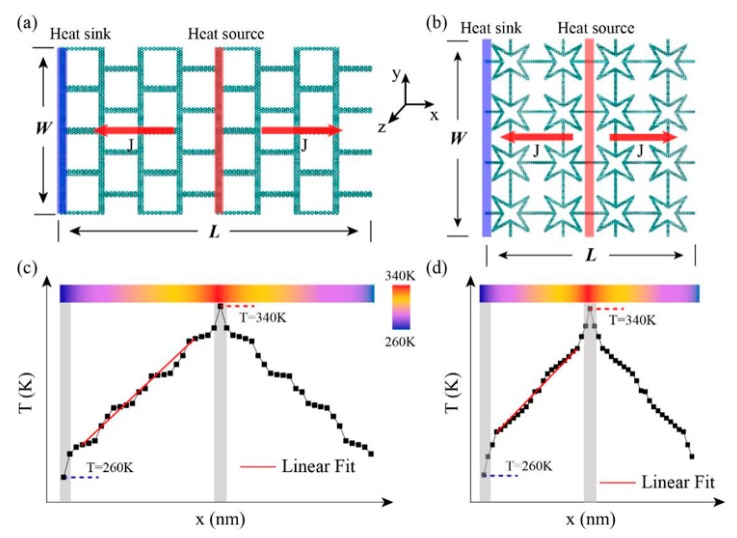
Schematic plot of the reverse non-equilibrium molecular dynamics method in calculating thermal conductivity of (**a**) SKG, and (**b**) QSS model. The average temperature distribution of SKG and QSS model are shown in (**c**,**d**).

Reverse non-equilibrium molecular dynamics simulations [24] were performed to calculate the thermal conductivity of the model. The model was divided into 50 slabs along the heat transfer (*x*) direction as approaching the equilibrium state. Figure 2a,b shows the 1st slab was assigned to be the heat sink while the 26th is the heat source, and the heat flux transfers from the heat source (hot region) to the heat sink (cold region). The heat flux transport direction is defined as the length direction (*L*) while the transverse direction is the width (*W*) direction. To generate a temperature gradient, the heat flux *J* is released/injected by exchanging the kinetic energies between the hottest atom and the coldest atom. The heat flux *J* can be obtained according to the following equations.

(1) $$J=\frac{\sum_{Nswap}\frac{1}{2}\left(mv_{h}^{2}-mv_{c}^{2}\right)}{t_{swap}},$$

where *N_swap_* is the amount of exchanging atoms pairs, *t_swap_* is the total time of exchanging kinetic energy, m is the mass of atom, and *v_h_* and *v_c_* represent the velocity of exchanging atoms, respectively. The temperature of each slab was collected and averaged over 3.0 ns to obtain temperature distribution when system reaches non-equilibrium steady state (after 1.5 ns).

The value of thermal conductivity was then calculated by using the Fourier’s law as

(2) $$\kappa =\frac{J}{2A\partial T/\partial L},$$

where *A* is the cross-sectional area of heat transfer and *∂T*/*∂L* denotes the temperature gradient after the system reaches non-equilibrium steady state (see Figure 2c,d). The factor 2 represents the fact that the heat flux transports in two directions away from the heat source. The thickness of model was assumed to be the interlayer equilibrium spacing of graphene (0.34 nm) [25,26].

## 3. Results and Discussion

The mechanical properties of these kirigami structures were investigated. To avoid any nonphysical strain hardening and spurious high bond forces [27,28], the cut-off distance was set to 0.2 nm [29,30,31]. When the system reached the equilibrium state, as the deformation-control method, the tensile loading was applied with a strain rate of 0.0001/ps by scaling all atomic coordinates accordingly for each 1000 steps under the NPT ensemble. When the tensile strain was applied in one direction, the structure was relaxed to 1 bar in the direction of perpendicular to the strain. Poisson’s ratio (*v*) is defined as *v* = −*ε_t_*/*ε_l_*, where *ε_t_* and *ε_l_* are the transverse strain and the longitudinal strain, respectively. The engineering stress is defined as:

(3) $$\sigma_{x}=\frac{1}{V_{0}}\frac{\partial U}{\partial \varepsilon_{x}},$$

where *U* is the strain energy, *V*_0_ is the initial volume of the system, and *ε_x_* is the loading strain. The atomic stress of individual carbon atoms in the graphene sheet is calculated according to the equation [11]:

(4) $$\sigma_{ij}^{\alpha }=\frac{1}{\Omega^{\alpha }}\left(\frac{1}{2}m^{\alpha }v_{i}^{\alpha }v_{j}^{\alpha }+\sum_{\beta =1,n}r_{\alpha \beta }^{j}f_{\alpha \beta }^{i}\right),$$

where *α* and *β* are the atomic indices; *m^α^* and *v^α^* denote the mass and velocity of atom *α*, *i* and *j* denote indices in the Cartesian coordinate system, and *r_αβ_* is the distance between atom *α* and *β*. The second term sums over all atoms and incorporates the contributions of kinetic energy, pairwise, and many-body interactions. The stress on each atom was averaged over the last latter 500 timesteps of the relaxation period. The global stress of the system was then obtained by averaging the stress on each atom over all.

**Figure 3:**
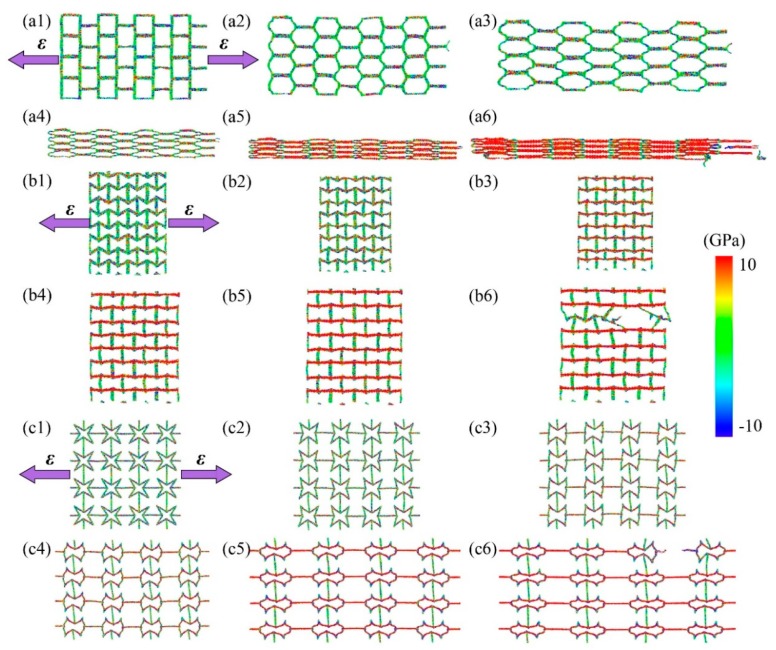
Configuration deformation and stress distribution of KGSs for system under tensile loading along the x-direction. (**a1**–**a6**) is process of the SKG’s model deformation and stress distribution under tensile loading. (**b1**–**b6**) is process of the RHH’s model deformation and stress distribution under tensile loading. (**c1**–**c6**) is process of the QSS’s model deformation and stress distribution under tensile loading.

**Figure 4:**
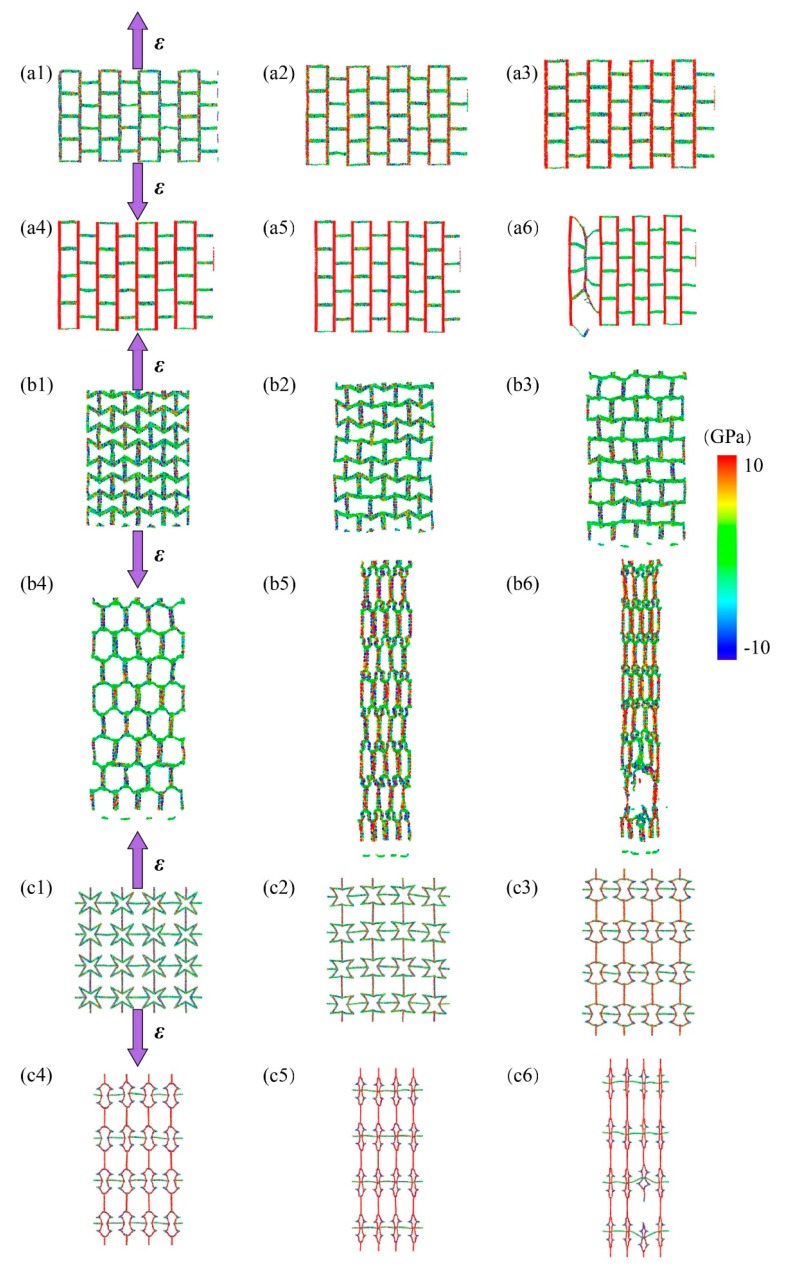
Configuration deformation and stress distribution of KGSs for system under tensile loading along the y-direction. (**a1**–**a6**) is process of the SKG’s model deformation and stress distribution under tensile loading. (**b1**–**b6**) is process of the RHH’s model deformation and stress distribution under tensile loading. (**c1**–**c6**) is process of the QSS’s model deformation and stress distribution under tensile loading.

All of these kirigami structure’s deformation and stress distribution under tension are plotted in Figure 3 (along x-direction) and Figure 4 (along y-direction), respectively. It can be found that the different tailoring structure induced diverse deform evolution and stress distribution field. For the SKG model, it shows explicitly anisotropic mechanical behaviors under tensile loading along x-and y-directions. When tension was conducted on SKG in x-direction, its system shrinked in the direction perpendicular to loading direction and it gathered together before system failure. While when it was stretched along the y-direction, tensile force was performed directly on y-parallel ribbons and it had barely geometry deformation in the tensile loading. For the RHH model, its dimension size in tensile perpendicular direction expanded (shrinked) under tensile loading along the x- (y-) direction. For the QSS model, it shows similar trends of stress, strain, and Poisson ratio as a function of strain in both x- and y-directions.

**Figure 5:**
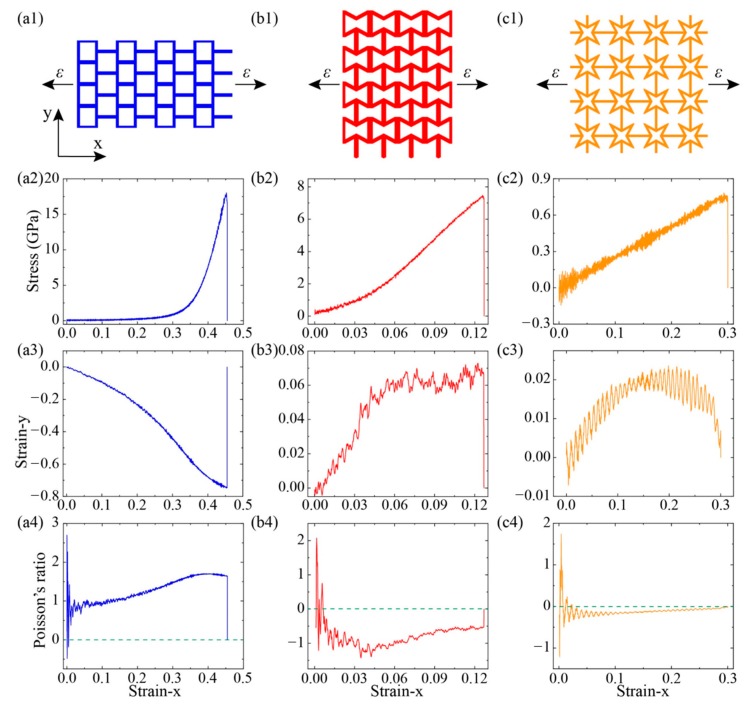
Mechanical properties of KGSs are explored by performing tensile loading along x-direction (**a1**–**c1**). The relationships of stress–strain (*σ*–*ε*) (**a2**–**c2**), strain–strain (*ε_x_*–*ε_y_*) (**a3**–**c3**), and Poisson’s ratio–strain (*v*–*ε*) (**a4**–**c4**) are plotted.

To further analyze the mechanical properties of three KGSs, the relationships of stress–strain (*σ*–*ε*), strain–strain (*ε_x_*–*ε_y_*), and Poisson’s ratio–strain (*v*–*ε*) of these models are shown in Figure 5. For the case of SKG model, the stress was almost zero at the initial elongation (strain <0.3). When the strain was over 0.3, the stress increased with strain sharply until its structure failed at a strain of 0.46. In comparison, the fracture strain for pristine graphene was 0.13 in armchair direction. The enhancement of fracture strain in SKG model was attributed to the geometry deformation under the tensile loading. This deformation process also reflected in the variation of strain perpendicular to the loading direction. As shown in Figure 5(a3), when strain-x <0.35, strain-y shrinked dramatically with loading along the x direction. It shows that in that period, tensile strain mainly induced geometry deformation. When the loading strain was over 0.35, strain-y changed slowly with increasing strain-x. At that period, tension mainly induced variation of the atomic bond-length and bond-angle in graphene. Thus, stress rose sharply with tension in this period. Figure 5(a4) shows Poison’s ratio *v* changed with tensile strain. At the beginning of loading, there was a large fluctuation of *v*-strain curve due to the kirigami structure starting to deform. Then *v* increased dramatically with strain until strain-x reached 0.35. It suggests that the system shrinks fast in the perpendicular direction. When strain was over 0.35, *v* reached a plateau (1.4) with further elongation. This is because the geometry deformation induced by strain was replaced by variation of atomic bond-length and bond-angle.

For the case of RHH model, its fracture stress and strain were 7.4 GPa and 0.127, respectively. Its stress–strain curve was smooth in the whole loading process and no jump-discontinuity point. It suggests that in the tensile loading process, the kirigami geometry deformation accompanies with the atomic lattice variation (bond stretching and angle change). Strain-y increased with strain-x along with some fluctuations (Figure 5(b3)) and strain-y was above zero thus its system expended in both the x and y directions under tensile loading along the x-direction. In the initial stage (strain-x < 0.04), strain-y increased sharply with strain-x. In this stage, the system obtained the minimum NPR’s value at strain-x = 0.036 (see Figure 5(b4)). Then strain-y increased slightly with further loading until strain-x at 0.127.

For the case of QSS model, its fracture strain and stress were about 0.3 and 0.77 GPa, respectively. The same as the RHH model, the kirigami geometry deformation accompanied with the slightly atomic lattice variation in the loading process. According to the strain-y and strain-x relationship (Figure 5(c3)), the system size in the perpendicular direction (y-direction) enlarged with strain-x when strain-x <0.15. It shows strong oscillation in strain-x and strain-y curve, which was attributed to the oscillation of system under tensile strain. Its oscillation amplitude was related to the strain-rate and it decreased with lower strain-rate. While it did not change the strain variation trend and structure deformation. Then strain-y decreased monotonously with further tensile loading. Strain-y was found to be larger than 0.0, which means that the system size in the y direction was greater than its initial size and its Poisson’s ratio value was negative (see Figure 5(c4)). All of these three kirigami models generate geometry deformation at the beginning of the tensile loading period due to the existence of tailoring vacancy.

**Figure 6:**
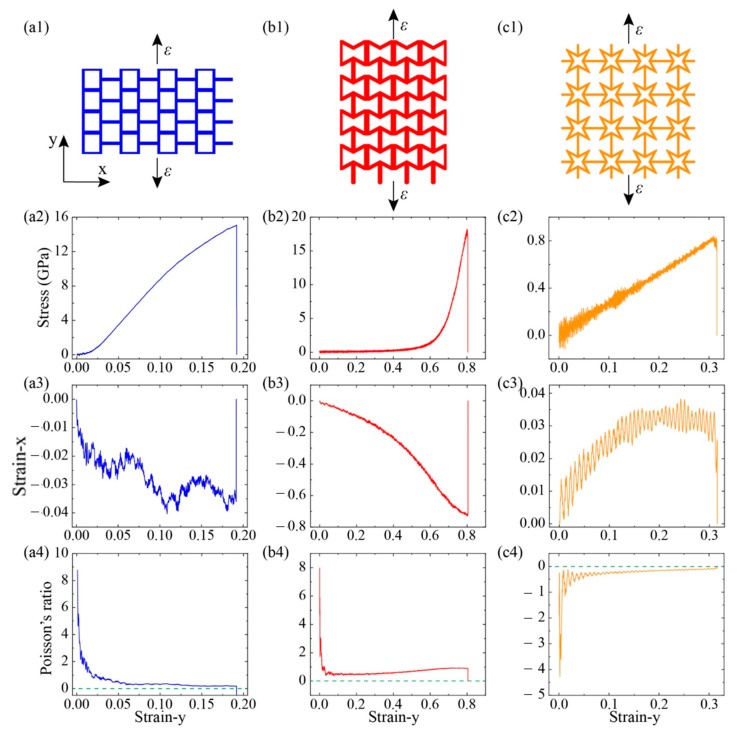
Mechanical properties of three KGSs are explored by conducting tensile loading along the y-direction (**a1**–**c1**). The relationships of stress–strain (*σ*–*ε*) (**a2**–**c2**), strain–strain (*ε_x_*–*ε_y_*) (**a3**–**c3**), and Poisson’s ratio–strain (*v*–*ε*) (**a4**–**c4**) are plotted.

Then we explored the mechanical properties of these kirigami structures by loading in y- directions (see Figure 6). For the case of graphene, its fracture stress at armchair and zigzag directions were 104 GPa and 127 GPa, respectively [32]. While for the KGS, its tailored structure played a dominant role in system deformation and its cutting shapes affected its anisotropic mechanical properties. For the SKG model, its fracture stress and strain were about 15 GPa and 0.19 along y-direction, respectively, which were smaller than its corresponding values along the x-direction (18 GPa and 0.46). That is because of tensile loading along the y-direction, it cannot release strain through geometry deformation (see snapshot of structural deformation in Figure 4). The fracture strain of SKG model in the y-direction was almost the same as pristine graphene in the armchair direction. System size of the x-direction shows a little variation in the loading process (its maximum deformation <0.04).

For the RHH model under tension along the y-direction, its stress did not increase explicitly at the initial stage until strain-y was over 0.60 (see Figure 6(b2)). It was attributed to strain mainly inducing geometry deformation at the initial stage. Therefore, its fracture strain reached 0.80 and its corresponding stress was only 17 GPa. The strain in the perpendicular loading direction, strain-x, decreased monotonously with strain-y, and it was less than zero in the whole loading process. Thus, its Poison’s ratio was positive in the RHH model under the tensile loading y-direction. While for the case of QSS model (see Figure 6(c2,c3)), its fracture strain and stress under loading along with the y-direction were 0.32 and 0.82 GPa, respectively. Its values were greater than those corresponding values under the x-direction loading, which were 0.3 and 0.77 GPa, respectively. The discrepancy of mechanical properties along the x- and y-directions was induced by graphene intrinsic mechanical anisotropic properties. Strain-x increased initially with strain-y and then reached a plateau (0.035) when strain was greater than 0.2 (see Figure 6(c3)). Comparing with its mechanical properties along the x-direction, we could see that their stress–strain, strain–strain, and *v*–strain curves were sharing the similar trends and values. That is because the cutting structure of QSS model was identified in both the x-and y-directions.

The mechanical properties of KGS were sensitive to its tailoring geometry and some tailored structures show high deformation in some directions under uniaxial tensile strain. For instance, the SKG shrinked by 73% in the y-direction when loading in the x-direction. The deformation parameters of kirigami structures were much greater than that of pristine graphene [32]. Moreover, some KGS exhibited the characteristics of a negative Poisson’s ratio. Considering that the thermal conductivity of material was inversely proportional to its cross-section area, the variation of system cross-section area affected its thermal conductivity. Moreover, as shown in Figure 5 and Figure 6, tension only induced geometry deformation in some KGS models, which had negligible effects on its phonon transport. To study the effects of structure deformation on thermal property, we calculated the thermal conductivity of the SKG and QSG in the x direction at various uniaxial tensile strains.

The SKG and QSS structures with dimensions of 30 × 20 nm^2^ and 50 × 50 nm^2^ were employed to study the strain effect on thermal conductivity of KGS. According to previous studies [11], thermal conductivity of graphene kirigami was independent of the system size for it is mainly dominated by short-range acoustic and optical phonons. We first calculated the thermal conductivity of models at strain-free at room temperature (T = 300 K). The thermal conductivity of SKG (2.9 W/mK) and QSS (1.2 W/mK) models were found to be much lower than that of pristine graphene sharing the same length of 30 nm (259.6 W/mK) and 50 nm (407.2 W/mK), respectively, but was in considerable agreement with previous studies by Wei et al. (5.1 W/mK) [11]. The reduction in thermal conductivity of KGS was attributed to the phonon scattering at the vacancy regions [33,34,35,36] and the decrease in real cross-section area [11].

**Figure 7:**
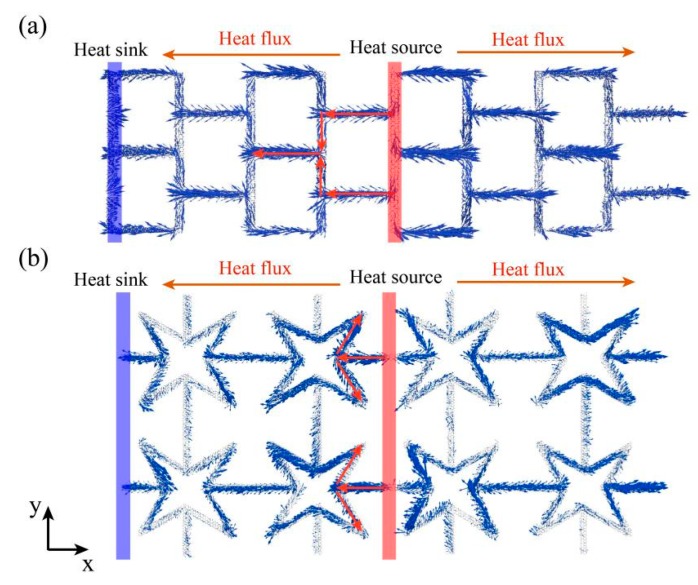
Spatial distribution of heat flux by vector arrows on each atom in SKG (**a**) and QSS (**b**) under the non-equilibrium steady state. The global heat flux transport directions are labeled with red rows with the heat source and heat sink are located at the middle and both ends of the model.

To clarify the reduction mechanism of the thermal conductivity and study the difference between KGS and pristine graphene in thermal transport, the spatial distributions of the heat flux on each atom in SKG and QSS were calculated. The micro heat flux was extensively used in describing the thermal transfer properties of low-dimensional materials. The atomic heat flux is defined as: *J_i_* = *e_i_v_i_* − *s_i_v_i_*, where *e_i_*, *v_i_*, and *s_i_* represents the energy, velocity vector, and stress tensor of atom *i*, respectively [36]. When the system reaches non-equilibrium steady state, the atomic heat flux will be calculated and averaged over 2 ns. As shown in Figure 7, the global heat flux labeled by red rows transferred from the heat source to the heat sink. The vector arrows labeled by blue rows show the migration and loss of heat flux as well as phonon scattering around the vacancy regions. Similar to the defect effect [33,34,35,36,37], the phonon scattering occurs when heat flux passes through a vacancy barrier, which results in the reduction of the thermal conductivity. Especially for the QSS model (see Figure 7b), the transfer direction of partial vector rows was opposite to that of global heat flux.

According to the research by Wei et al. [11], the reduction of the thermal conductivity of KGS was determined by three main factors, i.e., decreasing effective area of heat conduction, phonon scattering in vacancy region, and the elongation of the heat path. The effect of these three factors on thermal conductivity can be expressed as:

(5) $$\kappa =\delta_{s}\delta_{a}\delta_{p}\kappa_{0},$$

where *κ*_0_ represents the thermal conductivity of pristine graphene in the same size and *δ*_s_, *δ*_a_, and *δ*_p_ represent the reduction parameters caused by phonon scattering, effective area, and heat path, respectively. The phonon scattering *δ*_s_ can be obtained from *δ*_s_ = *κ*/(*δ*_a_ × *δ*_p_ × *κ*_0_). The values of these factors are shown in Table 1. We can see that the values of *δ*_s_ was one magnitude lower than that of *δ*_a_ and *δ*_p_, which indicates that phonon scattering was the dominant factor for the reduction of the thermal conductivity. Moreover, it has been reported that the parameter of heat flux path plays the main role in the reduction of the thermal conductivity of KGS in large system [11].

**Figure 8:**
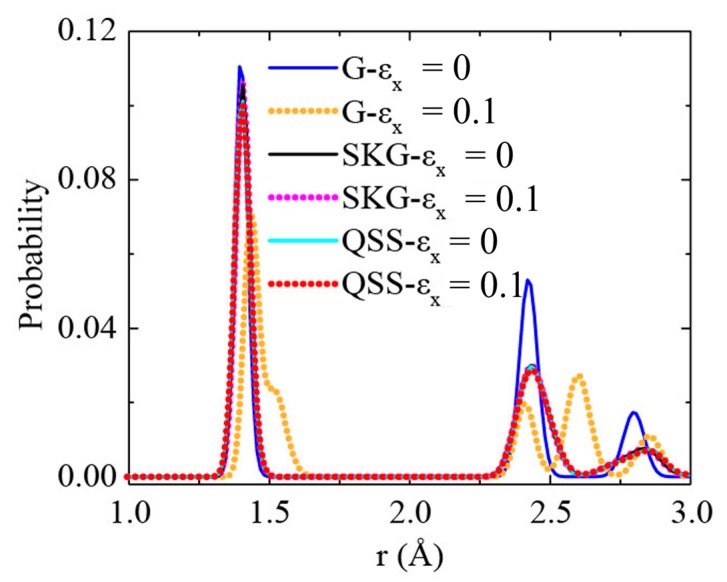
The radial distribution function of carbon atoms of graphene (G), SKG, and QSS models at strain-free and strain at 0.1. *ε_x_* represents the uniaxial tensile strain in the x-direction.

We computed the radial distribution function (RDF) of the SKG and QSS models to get the atomic deformation. The results of graphene were also plotted for comparison (see Figure 8). The first peak at 0.14 nm was observed in graphene, SKG, and QSS at the strain-free state. It suggests the C–C bond length was 0.14 nm in average [38,39].

The first RDF peak of graphene shifted to 0.143 nm at a uniaxial tensile strain of 0.1 in the x-direction, which means that the bonds of graphene were stretched. In contrast, the RDF of SKG and QSS show little difference between *ε_x_* = 0 and *ε_x_* = 0.1. It indicates that tensile strain barely induced C–C bond stretching but resulted in kirigami structure deformation (Figure 3 and Figure 4).

**Figure 9:**
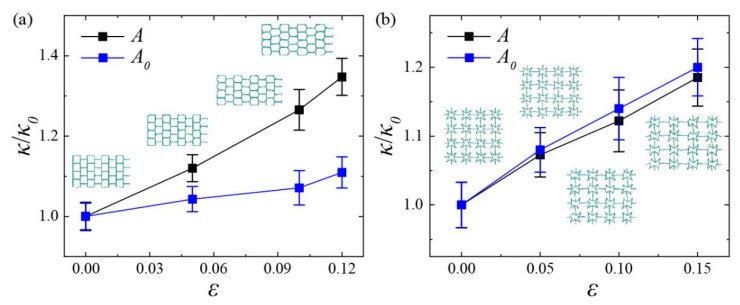
Relative thermal conductivity of (**a**) SKG and (**b**) QSS models in the x direction as a function of uniaxial tensile strain. *A* (*A*_0_) represents actual (initial) cross-section area with (without) considering the variation of lateral area.

In Figure 9, we show the thermal conductivity of SKG and QSS models in the x direction at various uniaxial tensile strains. To analyze the effect of cross-section area of models on thermal conductivity, the thermal conductivity was compared with (*A*) and without (*A*_0_) considering lateral cross-section area variation, respectively. All the values were normalized by the thermal conductivity (*κ*_0_) at strain-free. The values of *κ*_0_ of the SKG and QSS models were 2.9 W/mK and 1.2 W/mK, respectively.

As shown in Figure 9a, the thermal conductivity of the SKG was found to increase monotonously with tensile strain. This phenomenon was exactly the opposite of pristine graphene with small size, the thermal conductivity of which is reported to decrease with increasing tensile strain due to phonon softening and phonon scattering [5,6]. According to the study on mechanical property in Figure 5(a2), the global deformation of the SKG within small strain (<0.3) was mainly affected by the geometry deformation and the little variations of bonds and angles could even be ignored. Therefore, the strain effect of thermal conductivity of the SKG was mainly attributed to geometry deformation rather than phonon scattering. For the KGS, its model length increased with tensile strain due to geometry deformation, thus the acoustic phonons with longer wave-length were involved with heat transfer [40,41]. Compared to the thermal conductivity of strain-free (*ε* = 0, *κ*_0_ = 2.9 W/mK), the actual thermal conductivity of SKG model (*κ* = 3.9 W/mK) along the x direction increased by 34.7% when the strain reached 0.12. This actual value was larger than the thermal conductivity calculated by initial cross-section area under the same condition (*κ* = 3.2). The large difference between *S* and *S*_0_ was attributed to the sharply variation of cross-section area of the SKG under tensile strain.

As shown in Figure 9b, the thermal conductivity of the QSS model under uniaxial tensile strain shows the same tendency with the SKG model. According to the stress–strain curve in Figure 5(c2), the lower stress (<1.0 Gpa) indicates that the variation of microscopic atomic structure was so slight that the effect of strain on phonon states could be ignored. Therefore, the increase in thermal conductivity of the QSS model also derived from the increasing model length in the heat transfer direction due to the structural deformation. In comparison to the thermal conductivity at strain-free (*ε* = 0, *κ*_0_ = 1.2 W/mK), the actual thermal conductivity (*κ* = 1.4 W/mK) of the QSS model increased by 18.5% when the strain reached 0.15. In particular, the actual thermal conductivity of the QSS model was found to be slightly lower than that calculated by its initial cross-section area, which was exactly in contrast to the SKG. The difference could be attributed to their different mechanical properties. As we described in Figure 5 and Figure 6, the QSS model shows characteristics of negative Poisson’s ratio in the x and y direction, it means that its lateral aspect will expand with longitudinal tension. Since the thermal conductivity was inversely proportional to the value of cross-section area, the increasing cross-section area would lead to the decrease in thermal conductivity.

Comparing with traditional materials, some KGSs show large deformation in lateral direction under uniaxial strain. The variation of cross-section area led to a significant effect on thermal conductivity of KGS, such as the SKG model (see Figure 9a). The variation of the cross-section area was determined by the lateral strain *ε_t_*, and its relationship with tensile strain *ε_l_*, which could be described by parameter Poisson’s ratio *v* = −*ε_t_*/*ε_l_*. Therefore, when the tensile strain *ε_l_* is conducted, one can directly obtain the corresponding lateral strain *ε_t_* of the material based on its Poisson’s ratio *v*, i.e., *ε_t_* = −*v* × *ε_l_*. The lateral system size *W* under tensile strain can be defined as:

(6) $$W=W_{0}\times \left(1+\varepsilon_{t}\right)=W_{0}\times \left(1-v\times \varepsilon_{l}\right),$$

where *W*_0_ represents the initial lateral size of the material. Then, the relationship between initial cross-section area *A*_0_ and deformed area *A* can be expressed as:

(7) $$\frac{A_{0}}{A}=\frac{W_{0}\times d}{W_{0}\times \left(1-v\times \varepsilon_{l}\right)\times d}=\frac{1}{\left(1-v\times \varepsilon_{l}\right)},$$

where *d* is the thickness of the material. We defined *κ*_0_ as the thermal conductivity of KGS without considering the variation of the system in the lateral dimension. Due to the inverse relationship between the cross-section area and thermal conductivity, the thermal conductivity *κ* at various strains can be obtained by:

(8) $$\kappa =\frac{1}{\left(1-v\times \varepsilon_{l}\right)}\kappa_{0}.$$

**Figure 10:**
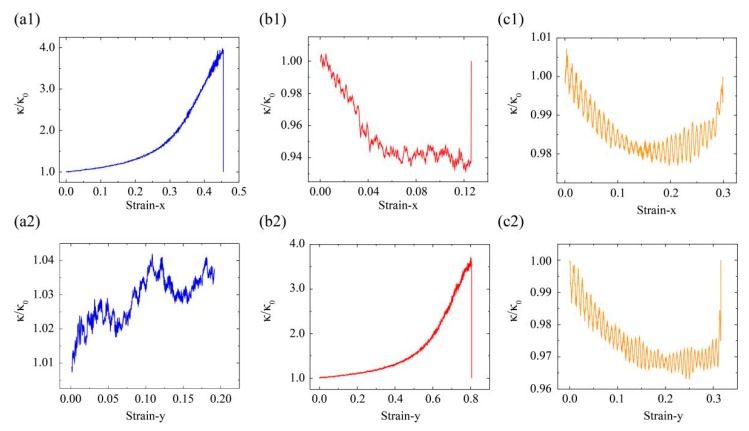
The variation of *κ*/*κ*_0_, 1/(1–*v* × *ε*_l_), in these GKS models (QSS, RHH, and SKG) under various uniaxial tensile strains along x- (**a1**–**a3**) and y-directions (**b1**–**b3**), respectively.

According to the obtained relationship between Poisson’s ratio and strain (*v*–*ε*) in Figure 5 and Figure 6, we show the variation *k*/*k*_0_ in these kirigami models under various tensile strains (see Figure 10). We can see that it could make almost four times the difference in thermal conductivity with and without considering lateral structural deformation in Figure 10(a1,b2).

## 4. Conclusions

In summary, we studied the strain engineering for thermal conductivity of tailored graphene kirigami models using molecular dynamics simulations. Our results show that tailoring geometry dominated the structural deformation of the system. It mainly influenced the thermal conductivity of the system by changing the lateral area, which varied inversely to its thermal conductivity. The thermal conductivity of square kirigami graphene and quadrilateral star structure increased by 34.7% and 18.5% when the strain reached 0.12 and 0.15, respectively. Moreover, we also found that it could reach four times the difference in thermal conductivity in our studied models with and without considering lateral area variation. Our results were explained by analyzing both the atomic scale, including heat flux and lattice deformation, and global structural deformation. Our findings provide useful guideline to use tailored graphene sheet in thermal management devices and thermoelectric materials.

## Figures and Tables

**Table 1:** The values of *δ*_a_, *δ*_p_, and *δ*_s_ of the SKG and QSS. *κ* and *κ*_0_ represents the thermal conductivity of KGS and pristine graphene.

Model	*κ* (W/mK)	*κ*_0_ (W/mK)	*δ*_a_	*δ*_p_	*δ*_s_
SKG (30 nm × 20 nm)	2.9	259.6 (30 nm × 20 nm)	0.348	0.635	0.051
QSS (50 nm × 50 nm)	1.2	407.2 (50 nm × 50 nm)	0.188	0.469	0.033
